# MARGRET M. AND PAUL B. BALTES AWARD PRESENTATION AND LECTURE

**DOI:** 10.1093/geroni/igac059.450

**Published:** 2022-12-20

**Authors:** Tamara Baker

## Abstract

The Margret M. and Paul B. Baltes Award Lecture will feature an address by the 2021 Baltes Award recipient, Laura B. Zahodne, PhD, of the University of Michigan. This session will also include the presentation of the 2022 Margret M. and Paul B. Baltes Award to recipient Eric S. Kim, PhD, of the University of British Columbia. The Margret M. and Paul B. Baltes Foundation Award in Behavioral and Social Gerontology recognizes outstanding early-career contributions in behavioral and social gerontology. The award is generously funded by the Margret M. and Paul B. Baltes Foundation.

